# Direct Effect of 10-Valent Conjugate Pneumococcal Vaccination on Pneumococcal Carriage in Children Brazil

**DOI:** 10.1371/journal.pone.0098128

**Published:** 2014-06-03

**Authors:** Ana Lucia Andrade, Yves Mauro Ternes, Maria Aparecida Vieira, Weslley Garcia Moreira, Juliana Lamaro-Cardoso, André Kipnis, Maria Regina Cardoso, Maria Cristina Brandileone, Iaci Moura, Fabiana C. Pimenta, Maria da Gloria Carvalho, Fabricia Oliveira Saraiva, Cristiana Maria Toscano, Ruth Minamisava

**Affiliations:** 1 Institute of Tropical Pathology and Public Health, Federal University of Goias, Goiania, Brazil; 2 Epidemiology Branch, Secretariat of Health of Municipality of Goiania, Goias, Brazil; 3 Pontifical Catholic University of Goias, Goiania, Brazil; 4 Laboratory of Bacteriology, Federal University of Goias, Goiania, Brazil; 5 Department of Epidemiology, School of Public Health, University of Sao Paulo, Sao Paulo, Brazil; 6 Adolfo Lutz Institute, Sao Paulo, Brazil; 7 Respiratory Diseases Branch, Centers for Disease Control and Prevention, Atlanta, Georgia, United States of America; 8 Faculty of Nursing, Federal University of Goias, Goiania, Brazil; Public Health England, United Kingdom

## Abstract

**Background:**

10-valent conjugate pneumococcal vaccine/PCV10 was introduced in the Brazilian National Immunization Program along the year of 2010. We assessed the direct effectiveness of PCV10 vaccination in preventing nasopharyngeal/NP pneumococcal carriage in infants.

**Methods:**

A cross-sectional population-based household survey was conducted in Goiania Brazil, from December/2010-February/2011 targeting children aged 7–11 m and 15–18 m. Participants were selected using a systematic sampling. NP swabs, demographic data, and vaccination status were collected from 1,287 children during home visits. Main outcome and exposure of interest were PCV10 vaccine-type carriage and dosing schedules (3p+0, 2p+0, and one catch-up dose), respectively. Pneumococcal carriage was defined by a positive culture and serotyping was performed by Quellung reaction. Rate ratio/RR was calculated as the ratio between the prevalence of vaccine-types carriage in children exposed to different schedules and unvaccinated for PCV10. Adjusted RR was estimated using Poisson regression. PCV10 effectiveness/VE on vaccine-type carriage was calculated as 1-RR*100.

**Results:**

The prevalence of pneumococcal carriage was 41.0% (95%CI: 38.4–43.7). Serotypes covered by PCV10 and PCV13 were 35.2% and 53.0%, respectively. Vaccine serotypes 6B (11.6%), 23F (7.8%), 14 (6.8%), and 19F (6.6%) were the most frequently observed. After adjusted for confounders, children who had received 2p+0 or 3p+0 dosing schedule presented a significant reduction in pneumococcal vaccine-type carriage, with PCV10 VE equal to 35.9% (95%CI: 4.2–57.1; *p* = 0.030) and 44.0% (95%CI: 14.–63.5; *p* = 0.008), respectively, when compared with unvaccinated children. For children who received one catch-up dose, no significant VE was detected (*p* = 0.905).

**Conclusion:**

PCV10 was associated with high protection against vaccine-type carriage with 2p+0 and 3p+0 doses for children vaccinated before the second semester of life. The continuous evaluation of carriage serotypes distribution is likely to be useful for evaluating the long-term effectiveness and impact of pneumococcal vaccination on serotypes reduction.

## Introduction

*Streptococcus pneumoniae* (pneumococcus) remains a major cause of serious bacterial infection worldwide, especially in infants living in developing regions [1], [2].

Children are the major reservoir of this pathogen. Pneumococcal carriage is highest during the first two years of life, and its frequency varies according to geographic location and socioeconomic pattern [3], [4]. Individual nasopharyngeal (NP) carriage or colonization is a prerequisite for pneumococcal disease and the only source of transmission [5].

Pneumococcal conjugate vaccines (PCV) have been available for over a decade but only recently were introduced into national immunization programs of developing countries. The first conjugate vaccinate available was 7-valent PCV (PCV7), which offered protection against 7 serotypes (4, 6B, 9V, 14, 18C, 19F, 23F). In 2010, PCV10 (including all serotypes in PCV7 plus 1, 5, 7F) was made available and PCV7 was replaced by PCV13 (including all serotypes in PCV10 plus 3, 6A, and 19A) [6]–[8].

Several studies have reported the effectiveness of PCV vaccines in reducing both invasive and non-invasive disease in children and adults [9]–[12]. Following PCV7 introduction, a reduction in pneumococcal carriage of PCV7 serotypes was described, as well as an increase in colonization by non-PCV7 serotypes [13], [14]. Evidence has indicated that overall pneumococcal carriage rates are unaltered after a period of PCV7 introduction, since decreases in vaccine serotypes are counteracted by increases in carriage of certain non-vaccine serotypes [14]–[19].

In 2010, PCV10 was introduced into Brazil’s National Immunization Program (NIP), with universal access for all children [20]. The early impact of vaccination on the reduction of pneumonia hospitalization in children in Brazil was recently reported [21]; however, the effectiveness of PCV10 on nasopharyngeal carriage has not yet been evaluated. Data describing the impact of PCV10 vaccination on carriage are scarce and to date no significant effects of PCV10 on carriage have been described [22], [23].

Although pneumococcal diseases are found in every age group [24], the primary reservoir of pneumococcus are children under five years old [2]; because of that, the effect of PCV10 vaccination on pediatric carriage and serotype distribution is critical.

The effect of PCV vaccination on carriage is of interest, since it could play a significant role in the overall vaccine impact on morbidity and mortality through minimizing vaccine serotype transmission to the non-vaccinated population.

Assessing PCV effect on carriage is not an easy task. Most studies have taken advantage of clinical trials to assess pneumococcal colonization as an outcome of interest. After vaccine introduction, one of the proposed methods to assess vaccine effectiveness on pneumococcal carriage is repeated sampling of the same individuals over time, which is expensive and invasive [24]. Recently, a cross- sectional study design was proposed to estimate vaccine effectiveness on carriage [24].

With the aim of assessing the direct effectiveness of PCV10 on pneumococcal carriage vaccine types, we conducted a cross-sectional study, during the first year of vaccination. We also investigated whether there was any difference of vaccine effectiveness between the different dosing schedules used in Brazil’s NIP during PCV10 introduction.

## Methods

### Study Design

A cross-sectional population-based household survey was conducted. We measured PCV10 vaccination effect by comparing vaccinated and unvaccinated individuals, all of whom are covered by the same voluntary vaccination program [25], [26]. The main outcome of interest was pneumococcal vaccine serotype carriage in children. The critical parameter of interest was PCV10 uptake assessing as vaccine doses. Prevalence of pneumococcal carriage was estimated. Children with carriage of vaccine serotypes (“cases”) were compared with children that were negative for pneumococcal carriage (“non-cases”). Non-vaccine serotype plus non-pneumococcal carriage were combined within the same category.

### Study Location

The study was conducted in Goiania Municipality (∼1,300 000 inhabs) in the Central West region of Brazil. Goiania is a highly urbanized municipality in Brazil, where there is a high rate of pneumococcal carriage and high vaccination coverage in infants [27]. The study period was from December/2010 to February/2011.

### Schedules Recommended by the Brazilian Immunization Program

PCV10 was made available free of charge to all children in Brazil through its introduction into the Public Healthcare System (*Sistema Único de Saúde,* SUS) during March to October of 2010 in all municipalities. Prior to that, PCV7 was available by the private healthcare system. In addition, SUS provided the vaccine for selected high risk individuals through its special immunization activities [20].

Three different schedules were used during PCV10 introduction in Brazil [20]:

- 3p+1: 3 primary doses for children between 6 weeks and 6 months of age (≤6 m), with an interval of two months between doses (minimum of 4 weeks) and a booster dose 6 months after the third dose.
- 2p+1 (catch-up): a schedule of two doses for children aged 7–11 months (7–11 m), with an interval of two months between doses (minimum of 4 weeks) plus a booster dose six months after the second dose.
- One single dose (catch-up): one dose for children aged 12–23 months (12–23 m).

For the country as a whole, the estimated vaccine coverage of a complete 3-dose series during the first year of the vaccine introduction was approximately 90% [28].

### PCV10 Introduction and Schedules Evaluated at the Present Study

PCV10 vaccine was introduced in Goiania Municipality in June 14^th^, 2010. The present study was conducted early during the first year of PCV10 introduction as routine immunization. The following PCV10 schedules were evaluated: 3p+0 (for children aged ≤6 m); 2p+0 (for children aged 7–11 m); and one catch-up dose (for children aged 12–23 m). Booster dose schedules (3p+1 and 2p+1) were not evaluated as the time period from vaccine introduction and case enrollment was short (6–8 months) and children enrolled did not have the opportunity to have been exposed to the booster dose.

### Study Population and Sample

For recruitment, the study targeted children of two age-groups: 7–11 months (7–11 m) and 15–18 months (15–18 m). The rationale behind that choice was to assess vaccine effectiveness considering the primary dose series as well as catch-up schedules used by NIP during the vaccine introduction period.

A list of all children aged 7–11 m and 15–18 m resident in Goiania Municipality was obtained from the National Information System of Live Births with information on each child’s gender, date of birth, address, and mother’s name. That list was sorted by gender, district of residence (as obtained with the address) and age. A systematic sampling process was put in place, so that a proportional stratified random sample of children was obtained. Sampled children were assessed through home visits.

Sample size was estimated at 1,287 considering a cross-sectional design and estimated percentage of pneumococcal carriage among non-vaccinated children of 58%. For the analysis of effectiveness, this sample size would provide the study with a power of 80% to detect as statistically significant (at the 5% level) a relative risk of 0.67 or more for a range of frequency of exposure among controls from 20% to 70% [29], [30]. Prevalence of pneumococcal carriage was obtained from a previous survey conducted in Goiania [27]. We estimated that about 65% would meet the study’s eligibility criteria; therefore a total of 1,906 children were initially sampled. Estimated relative risk was calculated considering data reported from PCV clinical trial among children 9 months of age [31].

### Inclusion and Exclusion Criteria

Sampled children were eligible to participate in the study if they lived in Goiania, had no prior antibiotic use during the previous seven days, and whose parent or legal guardian approved their child’s participation in the study with supplied informed consent signature.

Exclusion criteria included absence of the child’s parent or legal guardian during the home visit, lack of vaccination card, failure to collect NP swabs, and receipt of pneumococcal vaccines from different manufacturers.

### Ethical Considerations

Written informed consent was obtained by the child’s legal parents/legal guardians. The protocol was approved by the Ethics Committee of the Federal University of Goias (protocol #145/2010).

### Data Collection

During home visits, a standardized questionnaire was administered to legal guardians of all children included in the study. Data on sociodemographic characteristics and factors potentially associated with PCV10 effectiveness on vaccine-type carriage such as day-care attendance, number of children living in the same household, mother’s schooling, and access to private health insurance were collected. PCV10 vaccination dates were obtained from vaccination cards. For 9.1% of children, vaccination cards were not available. Thus, vaccination dates were obtained from Goiania’s Vaccination Online Database managed by the Municipal Data Processing Company, which is a comprehensive database, with high completeness, and updated on time by vaccination workers at the moment of vaccine administration in vaccination services.

### Nasopharyngeal Specimen Collection

Nasopharyngeal (NP) swabs were collected from all children included in the study by trained research assistants during home visits. Children were swabbed only once. Specimens were collected with flexible perinasal calcium alginate swabs (Fisherbrand, Fisher Scientific, Pittsburg, PA), which were placed into eppendorf tubes containing skim milk/tryptone/glucose/glycerol (STGG) transport medium [3]. The wired portion of the swab was cut at the top level of the tube, allowing the calcium alginate portion of the swab to drop into the vial. The samples were sent immediately after collection to the Bacteriology Laboratory of the Federal University of Goias, Brazil, vortexed to disperse the organisms from the swab, and frozen at −80°C.

### Specimen Processing

Cultures for *S. pneumoniae* were performed for all STGG samples. The frozen vials containing the NP swabs in STGG were thawed at room temperature and then vigorously vortexed for 20–30 s. A 200 uL aliquot was inoculated into 6 ml of THY broth (5 ml Todd-Hewitt broth with 0.5% yeast extract plus 1 mL of rabbit serum) for enrichment culture and incubated for 6 h at 37°C in a 5% CO_2_ incubator for conventional culture on a blood agar plate [32]. Alpha hemolytic colonies were tested for optochin susceptibility and bile solubility. Colonies with different morphologies from each sample were analyzed separately. Pneumococci confirmed isolates were frozen and sent to the *Streptococcus* Laboratory, Centers for Disease Control and Prevention (CDC) for serotyping by Quellung reaction with CDC-prepared antisera. Non-typeable (NT) pneumococci isolates were tested for the presence of 40 different capsular biosynthetic loci by conventional multiplex PCR (cmPCR), with 8 sequential, that identify a total of 22 serotypes and 18 small serogroups (see http://www.cdc.gov/ncidod/biotech/strep/pcr.htm for latest updates), as previously described [32].

### Definitions

*Age-groups at 1^st^ PCV10 dose:* we used the date of birth and the date at enrollment to assign children to each age-group. Children ages were retrospectively calculated based on the date of the first received PCV10 dose. For unvaccinated children, ages were retrospectively calculated based on the date of PCV10 introduction in Goiania Municipality (June, 14^th^, 2010). Children were then accordingly classified into three age-groups: ≤6 m; 7–11 m and 12–18 m, since each age-group had a different recommended number of doses (3p; 2p and one single catch-up), during the first year of PCV10 introduction.

*PCV10 dosing schedules*: Children were categorized according to the age-group at 1^st^ PCV10 dose and number of doses administered:

- Unvaccinated: Children who did not receive any dose or those who received only one dose before 12 months of age (for which, there was a recommendation of two or three doses). The rationale behind this assumption based on results of efficacy trials in Hib vaccine on carriage which showed that high serum anti-capsular type are needed for prevention of mucosal colonization [33].
- 2p+0: children who received two doses at any time during the first year of life;
- 3p+0: children who received three doses at any time during the first year of life;
- One catch-up dose: children who received one dose at or after 12 months of age.

In order to differentiate the booster dose from primary doses, we estimated the interval between doses; any dose administered with an interval of at least 6 months was considered a booster dose.

*Pneumococcal carriage*: children who had a positive NP swab culture for *S. pneumoniae*. Children were classified into the following pneumococcal carriage categories:

1. PCV10 serotype pneumococcal carriage (vaccine-type): children who were culture-positive for any of the pneumococcal serotypes 1, 4, 5, 6B, 7F, 9V, 14, 18C, 19F, or 23F;
2. Non-vaccine serotype pneumococcal carriage (non-vaccine type): children culture-positive for any serotype other than the ones included in PCV10;
3. non-pneumococcal carriage: culture-negative for pneumococci (regardless of culture results for other bacteria).

### Data analysis

Statistical analyses were performed using the STATA software, version 12.0 (Stata Corp, College Station, TX). The distribution of children according to age-group at recruitment and at 1^st^ PCV10 dose was compared.

Prevalence of pneumococcal carriage among children was estimated considering number of children colonized by *S. pneumoniae* in the numerator, and number of children surveyed for whom NP swabs were collected and processed in the denominator.

Pneumococcal serotypes identified by Quellung reaction were described (including those present in PCV10 and PCV13 composition). The main outcome of interest (exposure variable) was pneumococcal carriage of PCV10 vaccine type. Unvaccinated and vaccinated groups were compared regarding the following variables potentially associated with colonization: age-group (at enrollment), gender, number of children living in the same household, mother’s schooling, and day-care attendance.

Rate ratio (RR) for NP pneumococcal vaccine-type carriage, and its respective 95% confidence interval, was estimated as the ratio between the prevalence of vaccine-types carriage in children exposed to different dosing schedules (2p+0; 3p+0; and one catch-up dose), being unvaccinated children the reference group. Children with missing isolate serotype results were excluded from the analysis.

Confounding variables related with both, vaccine uptake and the outcome of interest (vaccine-type carriage) were included into the multiple regression model to estimate adjusted RR. Poisson regression with robust variance estimator was fitted to adjust the RR for confounding variables. For this investigation, variables associated with crowding (day-care attendance, and number of children in household), and mother’s schooling were identified as confounder; in addition, age (continuous variable) was also entered into the model.

PCV10 vaccine effectiveness (VE) on vaccine-type carriage was calculated using the adjusted RR and defined as the percentage reduction in the risk of pneumococcal vaccine-types in vaccinated children as compared with unvaccinated children as follows [24], [34]:
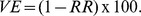



VE was reported with 95% confidence intervals (95%CIs). Statistical significance of VE was established if the lower limit of the 95% CI around VE was greater than 0 (zero). Table 1 displays the categories of interest used to assess the RR and VE on PCV10 vaccine-type carriage.

**Table 1:** Categories of exposure and outcome of interest considered for the estimation of rate ratio and vaccine effectiveness.

PCV10 dosing schedule	Pneumococcal carriage		Non-pneumococcal carriage†	Total of children surveyed
	PCV10 vaccine-type	Non-vaccine type		
Vaccinated (3p+0, 2p+0, one catch-up dose§)	a	b	N_1_– (a+b)	N_1_
Unvaccinated	c	d	N_2_– (c+d)	N_2_

§ Administered only for children 12 months of age or older.

† No culture for any other bacteria.

a+b+c+d  =  number of pneumococcal carriage, vaccine type and non-vaccine type.

c+d  =  number of unvaccinated children with pneumococcal carriage.

a+b  =  number of vaccinated children with pneumococcal carriage.



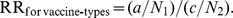



Therefore, the vaccine effectiveness VE for vaccine-types can be estimated as.
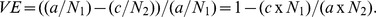



## Results

From 1,906 children whose households were visited, 1,287 (67.5%) met the inclusion criteria and were included in the study. Among the 1,287 children enrolled, 707 (54.9%) were aged 7–11 m and 678 (52.7%) were male. Dosing schedules included 404 children (31.4%) who received 3p+0; 372 (28.9%) received 2p+0; 291 (22.6%) received a single dose, and 220 (17.1%) were not vaccinated. Seventy children (5.4%) attended day-care and 832 (64.6%) used the public health insurance exclusively.

The distribution of children between age-group at enrollment and age-group at 1^st^ PCV dose is displayed on Table 2. The majority of children enrolled (53.7%) received the 1^st^ dose of PCV10 at the first semester of life.

**Table 2:** Distribution of children within age-groups according to age at enrollment and age at first dose, Goiania, Dec/2010 Feb/2011.

Age-group at 1^st^ PCV10 dose§	Age-group at enrollment‡		Total n (%)
	7–11 m n (%)	15–18 m n (%)	
≤6 m	662 (93.6)	29 (5.0)	691 (53.7)
7–11 m	45 (6.4)	240 (41.4)	285 (22.1)
12–18 m	0	311 (53.6)	311 (24.2)
Total	707 (100.0)	580 (100.0)	1287 (100.0)

‡ That is, the age-group at the moment of nasopharyngeal specimen collection.

§ Unvaccinated children were classified according to age at PCV10 introduction into National Immunization Program.

A total of 528 children were positive by pneumococcal culture yielding an overall prevalence of pneumococcal carriage of 41.0% (95% CI: 38.4%–43.7%). Prevalence of pneumococcal carriage for children aged 7–11 m and 15–18 m was 41.7% and 40.2%, respectively (*p* = 0.573). Of the 528 pneumococcal isolates, 494 (93.6%) were tested by Quellung reaction. Of these, 179 (36.2%) carried PCV10 vaccine-types.

**Figure 1:**
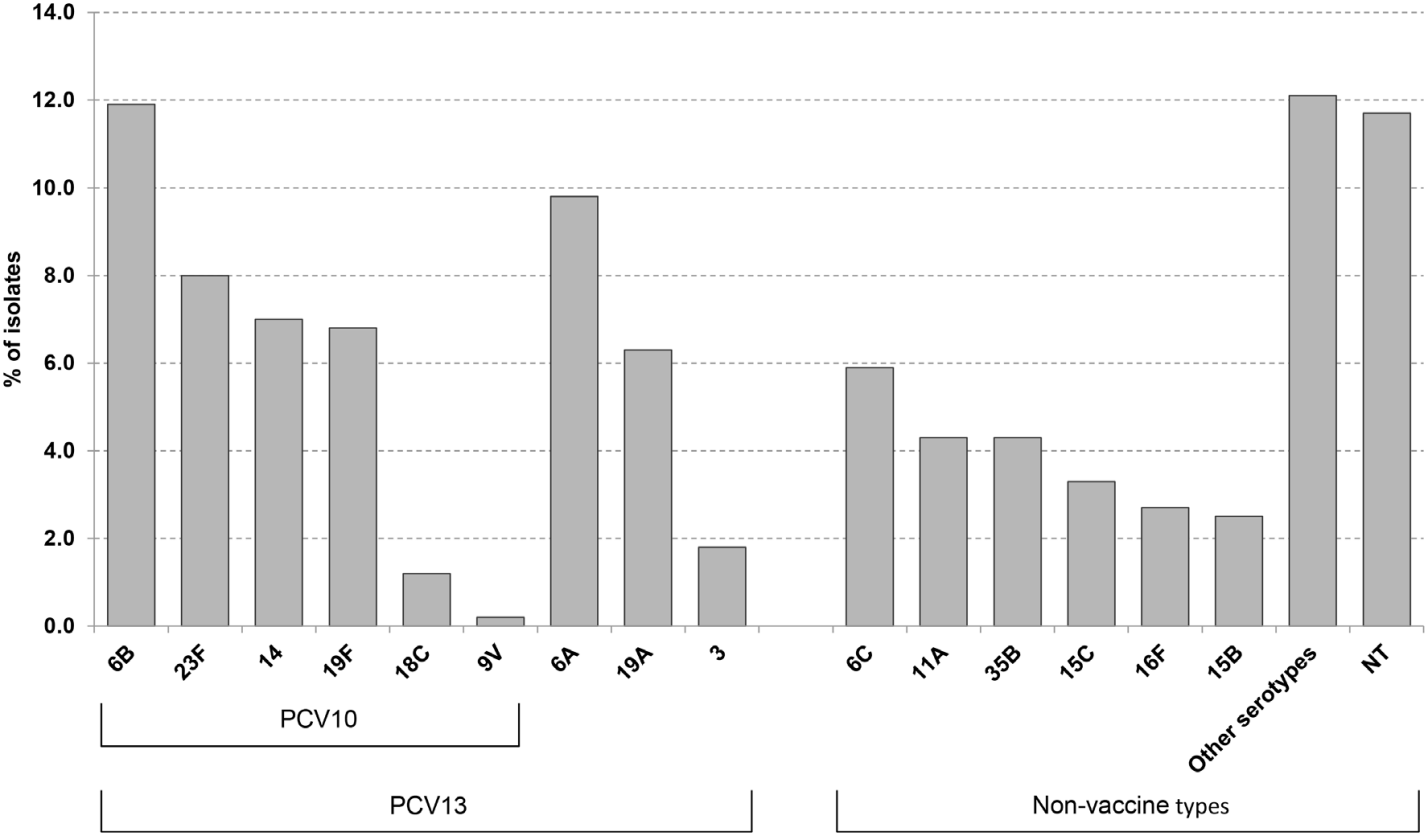
Distribution of pneumococcal serotypes carriage in children 7–18 months of age. Goiania, December/2010-February/2011. Other serotypes: 9A, 9N, 10A, 15A, 17F, 18A, 20, 21, 22F, 23A, 23B, 24F, 25A, 28A, 29, 31, 33F, 34, 35F.

Overall, 35 serotypes were identified among the 494 isolates tested, with the proportions of serotypes covered by PCV10 and PCV13 estimated at 36.2% and 53.0%, respectively. As seen in Figure 1, vaccine serotypes 6B (11.6%), 23F (7.8%), 14 (6.8%) and 19F (6.6%) were the most frequently observed, whereas PCV10 non-vaccine serotypes 6A (9.8%) and 19A (6.3%) were found in slightly higher proportions compared to 6C (5.9%), 35B (4.3%), 11A (4.3%) and 15C (3.3%). Thirty-four (6.6%) pneumococcal isolates could not be retrieved for serotyping. Simultaneous colonization with at least two pneumococcal serotypes was found in 17 (3.3%) children, six of which had a NT pneumococcal strain identified. Sixty (11.4%) of the non-serotypeable isolates were negative for capsular biosynthetic loci (positive *cps*A control as well as serotype-specific *cps* loci) corresponding to 40 serotypes or small serogroups. NT isolates (by both culture and by cmPCR) were observed in a higher proportion from children aged 15–18 months (59.4%) compared with children aged 7–11 months (40.6%) (*p* = 0.015). Serotypes 1, 5 and 7F were not detected.

The characteristics of 1,287 children according to vaccination status are shown in Table 3. Unvaccinated and vaccinated groups differed (*p*<0.05) in relation to number of children in household, mother’s schooling, and pneumococcal nasopharyngeal carriage.

**Table 3:** Characteristics associated with unvaccinated and vaccinated children with PCV10.

Variables	Unvaccinated†	Vaccinated‡	*p* value
	yes (%)	no (%)	
Pneumococcal nasopharyngeal colonization			
Yes	113 (51.4)	415 (38.9)	0.001
No	107 (48.6)	652 (61.1)	
Age-group at enrollment			
7–11 months	114 (51.8)	593 (55.6)	0.308
15–18 months	106 (48.2)	474 (44.4)	
Gender			
Male	109 (49.5)	569 (53.5)	0.306
Female	111 (50.5)	498 (46.7)	
No. of children<10 years old in household			
≥3	56 (25.6)	116 (10.9)	<0.001
≤2	163 (74.4)	951 (89.1)	
Mother’s schooling			
≤8 years	96 (43.8)	286 (26.9)	<0.001
>8 years	123 (56.2)	777 (73.1)	
Day-care attendance			
No	210 (95.5)	1006 (94.4)	0.519
Yes	10 (4.5)	60 (5.6)	
Total	220 (17.1)	1067 (82.9)	

† Defined as children who did not receive any dose at all (n = 96) or 1 dose before 12 months of age (n = 124).

‡ Defined as children who received 2 (n = 365) or 3 (n = 397) doses before 12 months of age or 1 cacth up dose >12 months of age (n = 291).

According to Table 4, there was a significant reduction of rate ratio from unvaccinated children to those who received 3p+0 dosing schedule for pneumococcal nasopharyngeal vaccine-type carriage (χ^2^ for trend = 5.08; *p* = 0.024).

**Table 4:** Rate ratio of PCV10 dosing schedule according to pneumococccal vaccine-type nasopharyngeal carriage.

PCV10 dosing schedules	Nasopharyngeal carriage of PCV10 vaccine-type		Total	Rate Ratio (95%CI)	*p* value
	Yes	No†			
Unvaccinated	40	171	211	1.0	
One catch-up dose§	44	239	283	0.820 (0.556; 1.210)	0.318
2p+0	46	320	366	0.663 (0.450; 0.978)	0.038
3p+0	49	344	393	0.658 (0.448; 0.964)	0.032
Total	179	1074	1253‡		

§ Administered only for previously unvaccinated children who received the vaccine at 12 months of age or older.

† Includes non-vaccine type carriage and non-carriage children.

‡ 34 isolates missing Quellung reaction results: unvaccinated (n = 9), one catch-up dose (n = 8), 2p+0 (n = 6), 3p+0 (n = 11).

Table 5 shows the results of PCV10 effectiveness for pneumococcal carriage vaccine-types in a multiple regression model. After adjustment for confounders, children who had received 2p+0 or 3p+0 dosing schedules presented a significant reduction in pneumococcal vaccine-type carriage, with PCV10 effectiveness equal to 35.9% (*p* = 0.030) and 44.0% (*p* = 0.008), respectively, when compared with unvaccinated children. In contrast, for children who received one catch-up dose, no significant reduction in vaccine-types was detected (*p* = 0.905).

**Table 5:** PCV10 effectiveness on pneumococcal vaccine-types carriage.

PCV10 dosing schedule	Multivariate Rate Ratio§ (95%CI)	% Vaccine effectiveness† (95%CI)	*p* value
Unvaccinated	1.0		
One catch-up dose‡	1.028 (0.650; 1.625)	–2.8 (–62.5; 35.0)	0.905
2p+0	0.641 (0.429; 0.958)	35.9 (4.2; 57.1)	0.030
3p+0	0.560 (0.365; 0.858)	44.0 (14.2; 63.5)	0.008

‡ Administered only for children 12 months of age or older.

§ Adjusted for no. of children in household, mother’s schooling, day-care attendance, and age (as continuous variable).

† Percentage based on results of multivariate analysis. Vaccine effectiveness: (1-RR)×100.

## Discussion

To the best of our knowledge, data describing the impact of PCV10 on carriage after its introduction in routine schedule of NIPs has not yet been reported. Brazil was the only country that used multiple schedules during the period of PCV10 introduction, with and without booster doses. Therefore, in this study, we were able to evaluate different dosing schedules before the administration of the booster dose (2p+0 and 3p+0, and also one catch-up dose). Hence, this investigation represented a unique opportunity to gather evidence of PCV10 effectiveness during a transition period, in which several dosing schedules were used for different age-groups, some of which were not used nor are currently being used by other countries.

The present study adds to the body of evidence describing PCV10 effects on NP carriage following different dosing schedules. Our data indicate that the primary series of either 3p+0 or 2p+0 dosing are effective in reducing PCV10 vaccine-type carriage. The overall vaccine uptake in Goiania reached high rates during the first 8 months of vaccination [28], which surely contributed to the rapid reduction of vaccine-serotypes within the age-group targeted by NIP for the primary schedules. Because no previous observational study has evaluated the effectiveness of 3p+0 or 2p+0 PCV10 dosing schedules on pneumococcal carriage, comparison of our findings is hampered. The majority of studies assessing PCV7 effectiveness against pneumococcal carriage was conducted at least 2 years after vaccine introduction. These studies also did not consider the number of doses [13], [14], [17], [18], [35]. In Colombia – Latin America, 2 years after PCV7 vaccination using a 2p+1 dosing schedule, an important reduction on vaccine-type carriage was observed among vaccinated children aged 12 to 18 months [36]. A recent systematic review of clinical trials assessing pneumococcal NP carriage provided evidence of decreased vaccine-type carriage for 2p+0, 2p+1, 3p+0 and 3p+1 schedules compared with no vaccine uptake, with the reduction greatest for the 3 primary dosing schedule [37].

As reported by other studies, we also observed a high prevalence of NT carriage isolates [27], [38], [39]. In addition to Quellung reaction for serotyping isolates, all NT pneumococci were tested by cmPCR. Considering that NT pneumococci recovered from carriage are often of non-encapsulated lineages, or can arise by mutations within *cps* genes or reduced gene expression, molecular tools are necessary for further characterization of NT strains [40], [41]. A considerable variety of serotype-specific *cps* loci were identified (n = 35) within NT isolates, with cmPCR types 19A, 19F, and serogroup 6 (26.6%) accounting for a large percentage of this isolate category (13% for 19A and 19F combined, 26.6% for serogroups 6A/6B and 6C/6D combined). A large number of isolates belonging to serogroup 6 was identified (26.6%), as well as serotypes 19A and 19F (13.0%). PCV13 cross-protection against the non-vaccine serotype 6C is a possibility [42], [43]. Considering that serotypes 6A and 19A are included within PCV13, the use of this vaccine could further decrease carriage of common disease-causing serotypes.

Some limitations should be taken into account when interpreting our results. Because this was an observational study, we measured associations between PCV10 introduction and changes in pneumococcal vaccine-type carriage. This does not reflect causality, although data on potential confounding variables was collected and considered in the analysis. We are also aware that some unmeasured factors such as viral infections, seasonal variations, vaccine coverage, and temporal trends could not be taken into consideration, as it was a short-term cross-sectional survey design. Nevertheless, cross-sectional studies of pneumococcal carriage could be a feasible, rapid and timely approach, when compared with follow-up studies for monitoring post-vaccination effects on pneumococcal serotype distributions. This methodology would be useful, mainly in settings where PCV has been recently introduced and/or vaccination coverage have not reached high rates [15], [44], [45].

Currently, the World Health Organization recommends the administration of 2 catch-up PCV10 doses at an interval of at least 2 months to unvaccinated children aged 12–24 months at the time of initial PCV10 vaccination [8]. Indeed, in our study, one single catch-up dose was not effective in preventing vaccine-type carriage for children 12 months or older. However, the possibility of lacking power should be considered, as the analysis was stratified by dosing schedule. This reduced the sample size, leading to a wide 95%CI of PCV10 effectiveness, which might have not allowed the detection of an eventual reduction in vaccine-type carriage for this schedule.

Unfortunately, since data collection took place only once and very early after vaccine introduction, there was not enough time to enroll children who received the booster doses, thus, preventing the assessment of 2p+1 and 3p+1 dosing schedule. Recent evidence has shown that the administration of a booster dose after two primary doses reduces pneumococcal spread from older children to other members of the community [46]. Further cross-sectional studies could further address this area.

In conclusion, we found that PCV10 was associated with high protection against vaccine-type carriage with a 3p+0 and 2p+0 dosing schedules for children vaccinated before the first year of life, soon after vaccine introduction into the routine immunization schedule. Anticipating that further changes in serotype distributions in all ages is likely to occur as a consequence of continued vaccination combined with herd effect, continued monitoring of carriage serotype distributions is valuable for evaluating the long-term effectiveness and impact of pneumococcal vaccination on reducing vaccine serotypes and on emergence of non-vaccine serotypes in Brazil.
